# Supplementary material for: Epidemiology of Antibiotic Resistant Pathogens in Pediatric Urinary Tract Infections as a Tool to Develop a Prediction Model for Early Detection of Drug-Specific Resistance
Source: Antibiotics (Basel). 2022 May 26;11(6):720. doi: 10.3390/antibiotics11060720 (PMC9220059; doi:10.3390/antibiotics11060720)
Supplement: Supplementary file 1 [file antibiotics-11-00720-s001.zip › antibiotics-1713513-supplementary.pdf]

Supplementary Figure S1. ROC curve and AUC of training and validation data sets

Amoxicillin-clavulanate /Cefixime	Ciprofloxacin/Cotrimoxazole
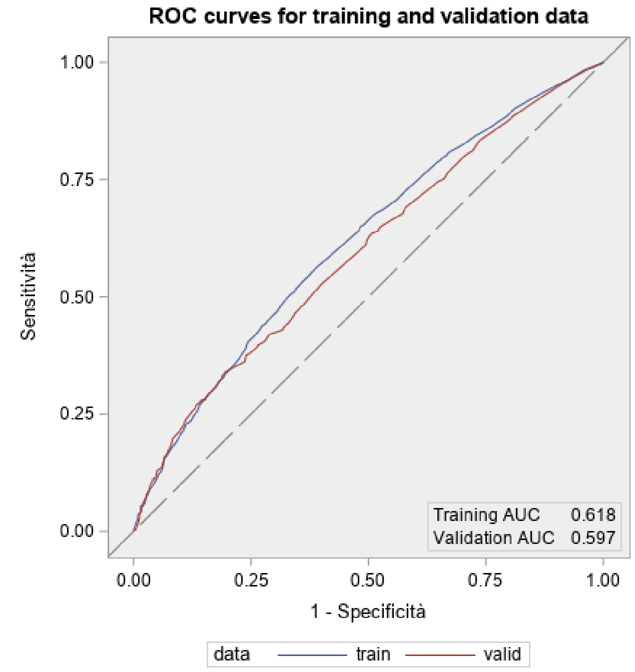	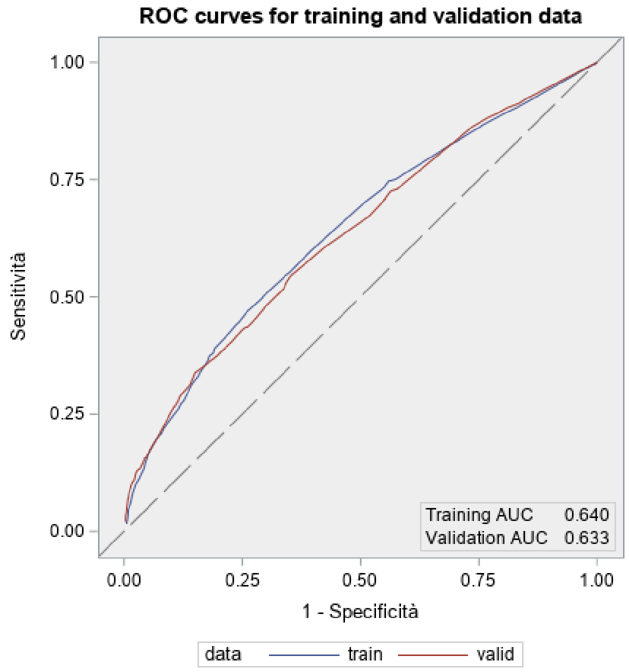

Supplementary Figure S2. Box plots of predicted probabilities and discrimination slope in the training and validation data sets

<table>
<tr><th>Amoxicillin-clavulanate /Cefixime</th><th>Ciprofloxacin/Cotrimoxazole</th></tr>
<tr><td>Training data set, discrimination slope =0.040</td><td>Training data set, discrimination slope =0.058</td></tr>
<tr><td>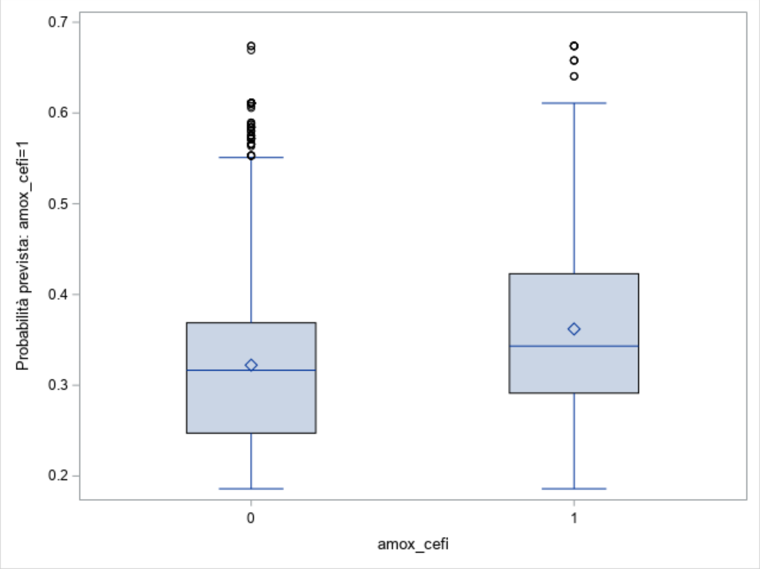
</td><td>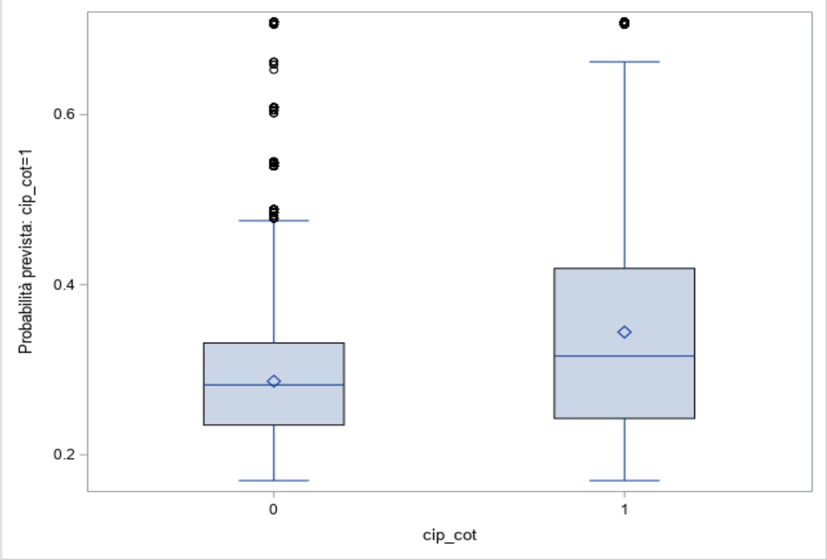
</td></tr>
<tr><td>Validation data set, discrimination slope =0.035</td><td>Validation data set, discrimination slope =0.057</td></tr>
</table>

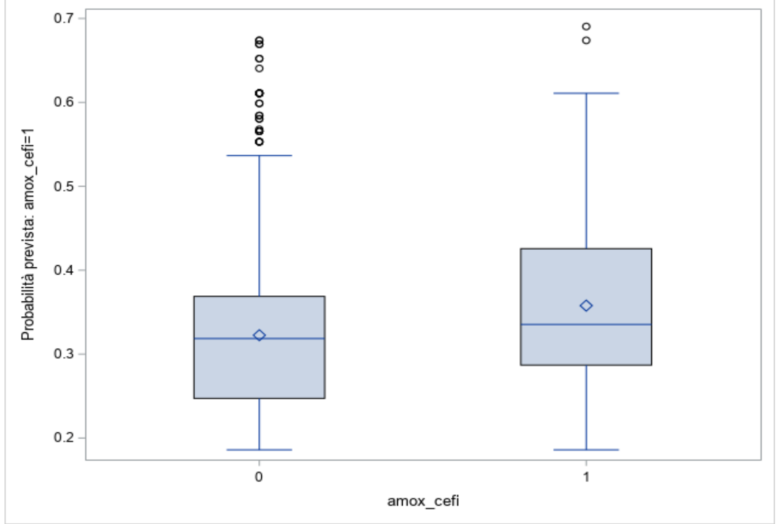

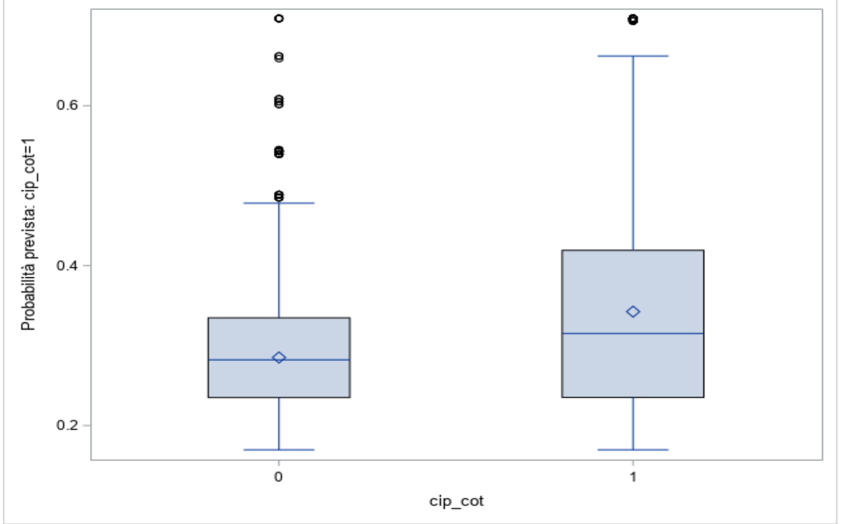

Supplementary Figure S3. ROC curve and AUC of 10-fold cross validations-internal validations

Amoxicillin-clavulanate /Cefixime

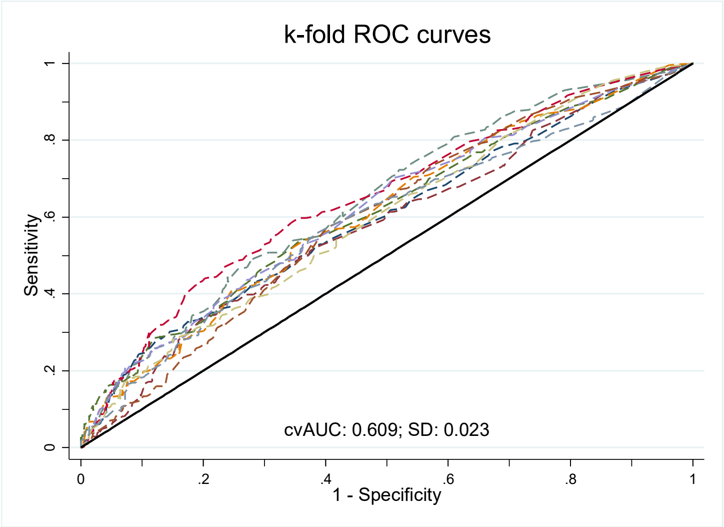

Ciprofloxacin/Cotrimoxazole

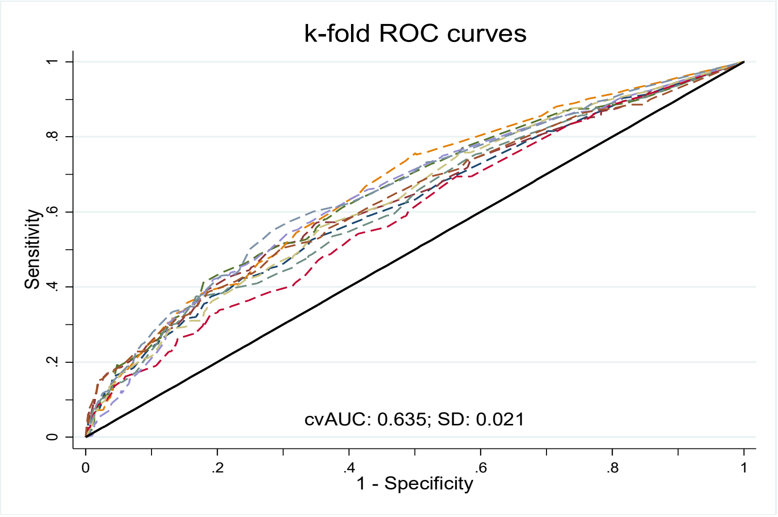

Supplementary Table S1. Distribution of 9449 bacterial pathogens isolated

Escherichia coli: n=4902 (51.9%), other *Enterobacteriales*: n=2694 (28.5%), other non-fermenting Gram-negatives: n=61 (0.6%), *Pseudomonas aeruginosa*: n=566 (6.0%), *Enterococcus* spp.: 995 (10.5%) and other Gram-positives: n=231 (2.4%)

Pathogens	n (%)	Group of pathogens
Escherichia coli	4902 (51.9)	*Escherichia coli*
Pseudomonas aeruginosa	566 (6.0)	*Pseudomonas aeruginosa*
Other *Enterobacteriales*	2694 (28.5)	Other *Enterobacteriales*
Citrobacter braakii	8	
Citrobacter farmeri	3	
Citrobacter freundi	69	
Citrobacter koseri	78	
Citrobacter species	3	
Citrobacter spp.	12	
Citrobacter youngae	3	
Enterobacter aerogenes	95	
Enterobacter asburiae	1	
Enterobacter cloacae	191	
Enterobacter hormaechei	3	
Enterobacter spp.	16	
Escherichia hermannii	1	
Klebsiella oxytoca	307	
Klebsiella ozonae	1	
Klebsiella spp	14	
Morganella morganii	109	
Proteus mirabilis	988	
Proteus spp.	44	
Proteus vulgaris/penneri	59	
Providencia rettgeri	3	
Providencia stuartii	2	
Raoultella ornithinolytica	1	
Salmonella spp.	3	
Serratia liquefaciens	2	
Serratia marcescens	39	
Serratia plymuthica	1	
Serratia spp.	5	
Klebsiella pneumoniae	633	
Other non-fermenting Gram-negatives	61 (0.6)	Other non-fermenting Gram-negatives
Achromobacter spp.	1	
Acinetobacter baumannii/calcoaceticus complex	9	
Acinetobacter junii	2	

**Pathogens**	**n (%)**	**Group of pathogens**
Acinetobacter lwoffii	1	
Acinetobacter spp.	3	
Pseudomonas fluorescens	2	
Pseudomonas pseudoalcaligenes	3	
Pseudomonas putida	7	
Pseudomonas spp.	29	
Stenotrophomonas maltophilia	4	
Other Gram-positives	231 (2.4)	Other Gram-positives
Staphylococcus aureus	61	
Staphylococcus saprophyticus	1	
Streptococcus agalactiae	169	
Enterococci	995 (10.5)	*Enterococcus* spp
Enterococcus casseliflavus	1	
Enterococcus faecalis	850	
Enterococcus faecium	99	
Enterococcus gallinarum	3	
Enterococcus spp.	42	

Supplementary Table S2. Characteristics of study population and culture reports of positive samples

**Total patients, n(%)**	**6207 (100.0)**
Sex, F	3136 (50.5)
Age at the 1st sample, median (IQR), min-max values, years	1.8 (0.4-6.8), 0-19
<=6 months	1685 (27.2)
>6 months	4522 (72.8)
Total numbers of bacterial pathogens isolated for patient, median (IQR), min-max values	1 (1-1), 1-55
1	4775 (76.9)
2	812 (13.1)
>2	620 (10.0)
**Total isolated pathogens[1]**	**9449 (100.0)**
Age at sample, median (IQR), min-max values, years	2.5 (0.6-8.3), 0-19
Department of admission	
Emergency	4679 (49.5)
Surgery/Orthopedics/Neurosurgery	1280 (13.5)
Nephrology	1044 (11.0)
Infectious Diseases	414 (4.4)
Neonatal or Pediatric ICU	383 (4.1)
Hematology/Oncology	353 (3.7)
Others	1296 (13.7)
Days from previous pathogens isolated, median (IQR), min-max values	94 (29-316) 0-4194
**Patients aged ≤6 months at the 1st sample**	**1685 (100.0)**
Sex, F	602 (35.7)
Total numbers of pathogens isolated for patient, median (IQR), min-max values	1 (1-2) 1-23
1	1238 (73.5)
2	239 (14.2)
>2	208 (12.3)
Department of admission	
Emergency	954 (56.6)
Hematology/Oncology/ Neonatal or Pediatric ICU /Others	389 (23.1)
Infectious Diseases	186 (11.0)
Surgery/Orthopedics/Neurosurgery	88 (5.2)
Nephrology	68 (4.0)

[1]9449 pathogen isolated in 9356 samples, 0.9% of samples had more than one isolate

Supplementary Table S3. Distribution of isolated pathogens resistant to different antibiotics. Proportions are number of resistant over number of tested strains

	**Resistant, n**	**Susceptible, n**	**% Resistant/Tested, (95% CI)**
***Escherichia coli*, n=4902**			
Amikacin	2	3000	0.07 (0-0.3)
Amoxicillin-clavulanate	1428	3350	29.9 (28.4-31.4)
Ampicillin	2685	1882	58.8 (57.1-60.5)
Cefixime	202	1689	10.7 (9.1-12.5)
Cefuroxime	336	3071	9.9 (8.7-11.2)
Cefotaxime	297	3442	7.9 (6.9-9.1)
Ceftazidime	273	4617	5.6 (4.9-6.4)
Ciprofloxacin	503	4391	10.3 (9.1-11.5)
Fosfomycin	43	4113	1.0 (0.6-1.6)
Gentamicin	344	4548	7.0 (6.2-8.0)
Meropenem	1	4891	0 (0-0.1)
Nitrofurantoin	22	4702	0.5 (0.3-0.7)
Cotrimoxazole	1518	3375	31.0 (29.4-32.7)
Piperacillin-tazobactam	468	4421	9.6 (8.6-10.6)
**Other *Enterobacteriales*, n=2694**			
Amikacin	22	1567	1.4 (0.8-2.3)
Amoxicillin-clavulanate	981	1579	38.3 (36.1-40.6)
Ampicillin	1864	518	78.2 (76.3-80.1)
Cefixime	205	881	18.9 (16.3-21.7)
Cefuroxime	398	1264	23.9 (21.8-26.3)
Cefotaxime	257	1829	12.3 (10.7-14.1)
Ceftazidime	295	2386	11.0 (9.7-12.5)
Ciprofloxacin	222	2454	8.3 (7.0-9.8)
Fosfomycin	423	1743	19.5 (17.7-21.5)
Gentamicin	419	2251	15.7 (14.1-17.4)
Meropenem	22	2658	0.8 (0.5-1.4)
Nitrofurantoin	973	449	68.4 (65.6-71.1)
Cotrimoxazole	605	2076	22.6 (20.6-24.6)
Piperacillin-tazobactam	373	2287	14.0 (12.6-15.6)
***Pseudomonas aeruginosa*, n=566**			
Amikacin	13	345	3.6 (2.1-6.3)
Ceftazidime	41	520	7.3 (4.6-11.5)
Ciprofloxacina	24	533	4.3 (2.5-7.2)
Fosfomycin	20	9	70.0 (46.4-85.1)
Meropenem	30	529	5.4 (3.6-7.8)
Piperacillin-tazobactam	46	509	8.3 (5.1-13.1)
**Enterococci, n=995**			
Ampicillin	79	867	8.3 (6.7-10.4)
Teicoplanin	6	951	0.6 (0.3-1.4)
Vancomycin	11	980	1.1 (0.6-2.0)
**Other Gram-positives, n=231**			
Ampicillin	23	0	100.0
Amoxicillin-clavulanate	2	10	16.7 (3.3-54.3)
**Other non-fermenting Gram-negatives, n=61**			

	Resistant, n	Susceptible, n	% Resistant/Tested, (95% CI)
Amikacin	2	39	4.9 (1.1-18.5)
Cefotaxime	11	2	84.6 (52.0-96.5)
Ceftazidime	7	40	14.9 (6.4-30.9)
Ciprofloxacin	10	49	16.9 (7.6-33.7)
Gentamicin	12	46	20.7 (10.0-37.9)
Meropenem	13	44	22.8 (11.6-40.0)
Cotrimoxazole	25	32	43.8 (30.1-58.6)
Piperacillin-tazobactam	6	41	12.8 (5.0-28.8)

Supplementary Table S4. Characteristics of patients with isolation of *Enterobacteriales* from urine samples

**Total patients, n (%)**	**5190 (100.0)**
Sex, F	2637 (50.8)
Age at the 1st sample, median (IQR), min-max values, years	1.6 (0.4-6.1), 0-19
<=6 months	1631 (31.4)
>6 months	3559 (68.6)
Total numbers of pathogens isolated for patient, median (IQR), min-max values	1 (1-2), 1-40
1	4065 (78.3)
2	657 (12.7)
>2	468 (9.0)
**Total strains**	**7596 (100.0)**
Age at sample, median (IQR), min-max values, years	2.3 (0.5-7.9), 0-19
0-6 months	1945 (25.6)
7 months-2 years	2255 (29.7)
3 -7 years	1498 (19.7)
>7 years	1898 (25.0)
Department of admission	
Emergency	4114 (54.2)
Surgery/Orthopedics/Neurosurgery	980 (12.9)
Nephrology	697 (9.2)
Infectious Diseases	319 (4.2)
Hematology/Oncology	283 (3.7)
Neonatal or Pediatric ICU	238 (3.1)
Others	965 (12.7)
Days from previous pathogens isolate, median (IQR), min-max values	106 (34-348), 0-4194

Supplementary Table S5. Distribution of 4902 *Escherichia coli* and 2694 other *Enterobacteriales* resistant to different antibiotics by age at sampling

	Age at sampling								
	0-6 months			>6 months			Total		
Antibiotics	R % (R/tested)	S	N	R % (R/tested)	S	N	R % (R/tested)	S	N
Amoxicillin-clavulanate	661 (34.8)	1239	45	1748 (32.1)	3690	213	2409 (32.8)	4929	258
Ciprofloxacin	94 (4.9)	1842	9	631 (11.2)	5003	17	725 (9.6)	6845	26
Cotrimoxazole	352 (18.2)	1585	8	1771 (31.4)	3866	14	2123 (28.0)	5451	22
Cefixime	118 (15.7)	635	1192	289 (13.0)	1935	3427	407 (13.7)	2570	4619
Ceftazidime	159 (8.2)	1780	6	409 (7.3)	5223	19	568 (7.5)	7003	25
Piperacillin-tazobactam	244 (12.6)	1686	15	597 (10.6)	5022	32	841 (11.1)	6708	47
Ceftazidime+Gentamicin, n=6648	65 (3.7)	1708	5	137 (2.8)	4720	13	202 (3.0)	6428	18
Piperacillin taz.+Gentamicin, n=6301	50 (3.0)	1600	6	126 (2.7)	4506	13	176 (2.8)	6106	19

R=resistant; S=susceptible
